# REIT Investment in US Skilled Nursing Facilities and the Quality and Cost of Care for Residents

**DOI:** 10.1093/geroni/igaf122.4341

**Published:** 2025-12-31

**Authors:** Mark Unruh, Jiebing Wen, David Stevenson, Hye-Young Jung, Robert Braun

**Affiliations:** Joan and Sanford I. Weill College of Medicine, Cornell University, New York, New York, United States; Weill Cornell Medicine, New York, New York, United States; Vanderbilt University, Nashville, Tennessee, United States; Joan and Sanford I. Weill College of Medicine, Cornell University, New York, New York, United States; Joan and Sanford I. Weill College of Medicine, Cornell University, New York, New York, United States

## Abstract

Real estate investment trusts (REITs) are publicly-traded or privately-owned companies that invest in the real estate of income producing properties. REIT investments in skilled nursing facilities (SNFs) have been associated with reductions in registered nurse staffing and worse health deficiency scores. However, the relationship between REIT investment in SNFs and resident outcomes is not known. We examined the association of REIT investment in SNFs with 4 measures of quality (30-day rehospitalization, discharge to the community, functional improvement, 180-day mortality), in addition to length-of-stay and Medicare costs for residents receiving post-acute care. A national database of REIT investments in SNFs was constructed and merged with Medicare claims and Minimum Data Set assessments for the period 2014 to 2021. In facility-level analyses, changes in outcome measures for SNFs with REIT investment were compared to those of for-profit SNFs without these investments using a difference-in-differences approach, adjusting for resident and facility characteristics. Among 9,936 SNFs in our sample, 1,174 (11.8%) had REIT investment. Following REIT investment, rates of discharge to the community worsened by 4.8 percentage points (95% CI: -8.2, -1.4), an 11.3% decrease compared to the baseline mean, relative to other SNFs. Estimates for other quality measures, SNF length-of-stay, and Medicare costs were not statistically significant. REIT investments may negatively impact SNF quality through lower rates of discharge to the community but are not associated with other quality outcomes or with Medicare costs for residents receiving post-acute care.

